# Food Fussiness Processes in Middle Childhood: Application of a Dual-Processing Model Using Measures of Temperament

**DOI:** 10.3390/nu17091489

**Published:** 2025-04-28

**Authors:** Jookyeong Lee, Alan Russell, Mohammadreza Mohebbi, Catherine G. Russell

**Affiliations:** 1 School of Exercise and Nutrition Sciences, Institute for Physical Activity and Nutrition (IPAN), Deakin University, Geelong, VIC 3220, Australia; georgie.russell@deakin.edu.au; 2 College of Education, Psychology and Social Work, Flinders University, Bedford Park, SA 5042, Australia; alan.russell@flinders.edu.au; 3 Biostatistics Unit, Faculty of Health, Deakin University, Geelong, VIC 3220, Australia; m.mohebbi@deakin.edu.au

**Keywords:** child, food fussiness, temperament, top–down bottom–up process, dual processing

## Abstract

Background: Analyses based on a dual-processing approach can contribute to a better understanding of the processes involved in food fussiness in children. This approach combines reactive or automatic avoidance responses together with regulatory processes, such as inhibitory control. Previous research has mainly focused on the avoidance response rather than both avoidance and regulatory control. Objective: The main purpose of the research was to investigate possible processes associated with food fussiness in children older than early childhood and into middle childhood (here, 5-to-12-year-olds) based on a dual-processing approach. Methods: The food fussiness subscale of the Children’s Eating Behavior Questionnaire (CEBQ) and the impulsivity, fear, shyness and inhibitory control subscales of the Children’s Behavior Questionnaire (CBQ) were used. Multivariable regression examined bottom–up/top–down temperament measures as components of the food fussiness process, with the main effects and interactions. ANOVA examined differences in the temperament measures for non-fussy, moderately fussy and severely fussy children. Results: The regression analysis showed that higher food fussiness was associated with lower impulsivity and lower inhibitory control. There also was a significant interaction between impulsivity and inhibitory control, suggesting that higher food fussiness for some children was associated with a combination of low impulsivity (more behavioral inhibition) and low inhibitory control. Conclusions: The results suggest that an analysis of food fussiness in terms of higher bottom–up avoidance in tandem with lower top–down inhibitory control is a helpful approach to the interpretation of the core processes involved in food fussiness in children. A better understanding of the fussiness processes can guide approaches to preventive interventions, including for parents of children with food fussiness.

## 1. Introduction

Fussy or picky eating in children is a concern for parents and health professionals [1]. It is often associated with underweight [1], not meeting dietary recommendations, especially for fruit and vegetables [1,2,3], and nutritional deficiency [4], and could be linked to later eating disorders [5,6]. Possible guidance for parents and professionals or in the design of preventive interventions is dependent on knowledge about child characteristics and processes associated with fussy eating. One emphasis in the research and theory about these characteristics and processes has been on children’s food avoidance or rejection [3,7], such as because of flavor or texture [8], and is sometimes linked to child characteristics such as negative affect, fear or shyness [9]. Less attention has been directed to children’s abilities to control or regulate the avoidance response to food in general or to specific foods. The present research, with 5- to 12-year-old children, was designed to investigate the possible relevance of an approach that combined both children’s avoidance responses and the control or regulation of these responses. This approach has been described as a dual-processing or dual pathway approach [10,11].

Another way of describing the dual-processing approach is in terms of a bottom–up, top–down model [12]. This model has been applied to the analysis of self-regulation in children [12], including appetite self-regulation (ASR) [13], especially in relation to children’s food approach tendencies and overeating [14,15]. This means that the two pathways or systems in a dual-processing approach are (a) a reactive or “bottom–up” approach or avoidant responses to prepotent stimuli and (b) regulatory or “top–down” processes such as inhibitory control [10,11,12,13,15,16,17,18,19].

The were two aims in the present study. The first was to investigate the application of a dual-processing approach to the examination of food fussiness in children beyond early childhood (here, aged 5 to 12 years) using temperament measures of bottom–up avoidance factors in the form of low impulsivity, fear and shyness, together with top–down inhibitory control. Greater food fussiness was expected to be associated with lower impulsivity, higher fear and higher shyness. An aspect of top–down regulation was measured by the temperament dimension of inhibitory control, with lower inhibitory control expected to be associated with food fussiness. The research has implications for the understanding of the core processes associated with food fussiness in children.

In addition, there was a secondary aim that involved the use of cut-off scores to report the incidence of food fussiness in children aged 5 to 12 years. This is relevant to questions about levels of food fussiness beyond early childhood and into middle childhood. It enables the investigation of the characteristics of children categorized as severely fussy and comparisons with other research, including the incidence of food fussiness in infancy and early childhood.

The article begins with a review of the literature on processes and child characteristics associated with food fussiness in children. The research undertaken is then explained, and the results presented. The discussion highlights the implications for the conceptualization of food fussiness in children and practical implications.

### Literature Review

The dual-processing approach and bottom–up, top–down model draw on cognitive and neuroscience perspectives of self-regulation [15,16]. Top–down processes pertain to effortful control that are served by cortical structures and the anterior cingulate cortex, while bottom–up reflects more automatic or reactive processes associated with subcortical structures [16]. This conception parallels other approaches to the analysis of self-regulation, including intuition and emotion versus reflection and analysis [17], thinking fast and slow [19], and impulsive versus inhibitory eating (such as resisting automatic responses to food cues) or mindful eating (such as awareness of food cues, hunger cues and attitudes to food) [18,20]. In the case of food fussiness, the dual-processing approach suggests that bottom–up food avoidance responses are not balanced by regulatory control processes [10,21].

Food fussiness in children pertains to the avoidance of both new and familiar foods and is characterized as a persistent and steady trait over time [22]. Food neophobia refers to rejection of new and novel foods and is therefore a sub-construct of food fussiness [23]. From an evolutionary perspective, fussiness, particularly the neophobia component, is an adaptive and useful trait in the avoidance of toxic and harmful foods and is therefore accepted as a normal behavior [24]. Food fussiness can become maladaptive if it is associated with less healthy diets [25]. Much of the research on food fussiness/picky eating and food neophobia has focused on infancy and young children [22]. Researchers have concluded that food fussiness peaks in later early childhood and declines by the age of 6 or in middle childhood (5 to 12 years) [26]. But for some children, fussiness persists into later childhood or recurs during middle childhood and adolescence [27], with fussiness possibly persisting into adulthood [28]. Research on the incidence of food fussiness in middle childhood has reported rates comparable to those in early childhood [29], but the factors relating to food fussiness and processes associated with fussiness may change with age [24,28,30,31,32].

Research on possible processes associated with food fussiness in children has examined the role of child temperament and related behaviors. Most of this evidence pertains to infancy and the preschool years [32,33] and has been directed to emotional dimensions of temperament and to approach-withdrawal [24,32,33,34]. Consequently, the emphasis has been on children’s tendencies to avoid or reject particular foods. A value in investigating food fussiness based on the dual-processing approach is that it gives attention to the role of reactivity together with regulatory control, which has not been a focus thus far.

In addition to measures of temperament, children’s age and sex have been investigated as factors associated with food fussiness [35]. However, most studies disregard the roles of age and sex and include them as peripheral demographics with no clarity about how children’s sex and age are associated with underlying processes and pathways of food fussiness [9]. The present research has the potential to contribute to the understanding of processes associated with age and sex differences in food fussiness through the application of the dual-processing approach.

## 2. Materials and Methods

### 2.1. Study Design and Sample Size

This study was cross-sectional. The aim was to recruit a large and diverse sample of children aged 5 to 12 years of age. The minimum sample size was estimated to be 1033. A sample size of 1033 achieves more than 90% power to detect a standardized effect size (i.e., R-squared) of 0.07 attributed to 4 independent variables namely impulsivity, fear, shyness, and inhibitory control using an F-test with a significance level of 0.05. The variables tested are adjusted for age with an R-squared of 0.06.

### 2.2. Participants

Participants were children aged 5–12 years and their parents/caregivers who visited a science museum (Spotswood, VIC 3015, Australia) in January 2020. The inclusion criteria were parents and their children 5 to 12 years old who could read and understand English. A total of 1357 children and their parents initially participated in the study, with 1188 children included in the final data analysis after excluding 169 participants due to missing age, sex or incomplete food fussiness measures. All participants provided written informed consent, and child assent was obtained verbally.

### 2.3. Demographic and Anthropometric Characteristics

Children’s age and sex were recorded from parent responses. The child’s biological sex was asked in a questionnaire form (i.e., what is your child’s sex?) with 4 response options of male, female, non-binary, and self-described (please specify-open-ended). For height and weight measures, children were asked to remove shoes, socks and heavy clothing. Standard procedures were then followed using a portable stadiometer (Seca213) (Seca, Hamburg, Germany) and a segmental body composition analyzer (TBF-300A) (Tanita Corporation, Tokyo, Japan), respectively. Height and weight were measured in units of centimeters and kilograms, respectively, with four and three significant digits, respectively. BMI (weight in kg/m^2^) was calculated based on the measured height and weight. BMI groups were created (underweight, healthy weight, overweight and obese groups) based on calculated BMI and then adjusted by children’s age and sex based on BMI cut-offs provided by the Australian Bureau of Statistics [36].

### 2.4. Food Fussiness

Food fussiness was measured using the Children’s Eating Behavior Questionnaire (CEBQ)-food fussiness subscale (6 items) [37]. Parents responded on a 5-point Likert-type scale with an option for “do not know”. Measured food fussiness was categorized into non-fussy, moderate and severe groups based on the cut-off values determined by a previous study [38]. That study validated the food fussiness subscale of the CEBQ against an interview-based psychometric measure (the Preschool Age Psychiatric Assessment) in 752 6-year-old Norwegian children using the non-parametric receiving operating curve (ROC) approach. Mean fussiness cut-offs with maximum sensitivity and specificity were 3.0 and 3.33 for the moderate and severe groups, respectively, which were applied to the present study. Mean scores < 3.0 indicate non-fussy, between 3.0 and 3.33 moderately fussy and >3.33 severely fussy [38]. Internal consistency (Cronbach α) of the food fussiness subscale was 0.75 in the current study.

### 2.5. Temperament

Child temperament was assessed with the Children’s Behavior Questionnaire (CBQ)- Short Form [39]. The inhibitory control, impulsivity, fear, and shyness subscales containing 6 items each (24 items total) were used, and the participants responded on a 7-point Likert-type scale with an option for “not applicable”. The scale has been found to have satisfactory internal consistency, criterion validity, longitudinal stability and cross-informant agreement for a sample of White, middle-to-upper socioeconomic children 4 to 6 years of age [39]. Higher scores indicate greater temperamental tendency with the score range of 1 to 7 (1—extremely untrue, 2—quite untrue, 3—slightly untrue, 4—neither true nor untrue, 5—slightly true, 6—quite true, 7—extremely true). Internal consistency (Cronbach α) was 0.76, 0.72, 0.73 and 0.76 for inhibitory control, impulsivity, fear, and shyness subscales, respectively. These levels are comparable to those reported as acceptable in the development of the scale [39].

### 2.6. Statistical Analysis

Prior to data analysis, data were visually inspected for serious departure from normality using graphs. There were a few extreme values in weight and height measures, which were excluded during data cleaning. There were missing values in weight, height and/or temperament measures, and these were treated as missing values and not included in data analysis. The outcome variable was food fussiness, while the explanatory variables were children’s characteristics (age, sex and BMI) and temperament domains (inhibitory control, impulsivity, fear and shyness). Food fussiness was analyzed as a continuous variable for correlations and linear regression analyses and as a categorical variable (derived from cut-off values used in screening research) for ANOVA comparisons of severely fussy children with non-fussy and moderately fussy children. Descriptive statistics (means ± standard deviations (mean ± SD)) were calculated for the children’s characteristics, fussiness scores and fussiness categories. One-way ANOVA and Tukey’s post hoc test was performed to examine differences in the means of children’s characteristics and temperament among the three fussiness groups. Differences were tested using the F-statistic. Spearman’s correlation coefficient (r) was used to identify linear relationships between the food fussiness score and children’s age. An independent sample *t*-test between food fussiness (continuous variable) and sex was used to test for differences in food fussiness scores between males and females. Pearson’s correlation coefficient (r) was used to examine linear relationships between food fussiness scores and children’s BMI and the temperament measures. From the correlation analysis, only age was significant and included in the multivariable regression [32,38]. Even though BMI was significantly correlated with food fussiness, it was not included in the regression model because BMI is an outcome of food fussiness, rather than a factor that could affect food fussiness processes [27,40]. Collinearity was assessed for the regression analysis by assessing variation inflation factor (VIF). Assessed VIF ranged between 1.02 and 1.51. Multivariable linear regression analysis was performed to investigate the association between food fussiness and the application of the dual-processing approach. Food fussiness was the dependent variable, age was included as a covariate, and inhibitory control, impulsivity, fear and shyness were the independent variables. A hierarchical (adding blocks of variables) regression approach was used. Age was entered as the first step, followed by the block of impulsivity, fear, shyness and inhibitory control. The third block involved interactions between inhibitory control and impulsivity, inhibitory control and shyness, and inhibitory control and fear. Two-way interaction terms between inhibitory control and fear; inhibitory control and shyness; inhibitory control and impulsivity were investigated in additional models. The results of the regression analysis were expressed in beta coefficients (β) and 95% confidence intervals (CI). Partial eta squared was used as a measure of effect strength (0.02: small effect; 0.13: medium; 0.26: large) [41]. Adjusted R-squared was reported as a measure of goodness of fit. Alpha level was set at 0.05. Stata Statistical Software version 18.0 [42] was used for the analysis. Alpha level was set at 0.05.

## 3. Results

### 3.1. Children’s Characteristics

The children’s anthropometric and sociodemographic characteristics are presented in Table 1. There were slightly more males than females in the sample. The mean age was 7.8 years. Nearly half the children were aged 5–7 years with smaller proportions aged 8–9 years and 10–12 years. The BMI groups showed that the majority (65.0%) of the children were of healthy weight with 6% in the obesity range. A significant difference was not found for children with and without BMI data according to age (t-statistic (1186) = −1.65, *p* > 0.05) and sex (*X*^2^ (1, 1188) = 0.86, *p* > 0.05).

### 3.2. Prevalence and Mean Scores of Food Fussiness

Overall, the mean fussiness score was 2.9 ± 0.9. Screening cut-offs were used to divide the sample into non-fussy, moderately fussy and severely fussy groups. The prevalence rates for these groups are shown in Table 2. More than half of the children were classified as non-fussy, with about one third as severely fussy and a smaller proportion were moderately fussy.

### 3.3. Children’s Characteristics by Fussiness Status

Mean scores for children’s age, BMI and the temperament measures were calculated for each fussiness group. ANOVA results with follow-up Tukey’s post hoc comparisons among the fussiness groups are presented in Table 3. A significant difference was not found for age and sex, but for all other variables in the table there were significant differences among the groups. The post hoc comparisons reported in Table 3 showed that BMI was lower in the moderate and severe fussiness groups compared to non-fussy children. Inhibitory control was lower in the severely fussy group compared to the moderately fussy and non-fussy children. Impulsivity was significantly lower for severely fussy children compared to non-fussy children. Fear was significantly higher in severely fussy children compared to non-fussy children and moderately fussy children. Shyness was also significantly higher in severely fussy children compared to non-fussy children. Statistically significant differences were not found for mean age and sex among the fussiness groups (*p* > 0.05). In summary, severely fussy children had lower BMI, and their scores were lower for inhibitory control, and impulsivity, and higher for fear and shyness.

### 3.4. Correlations Between Food Fussiness and Children’s Characteristics

Correlations for food fussiness and children’s characteristics and temperament are shown in Table 4. Higher food fussiness was correlated with lower age, BMI, impulsivity and inhibitory control and higher fear and shyness. These correlations are consistent with the results reported in Table 3. There was no evidence of a significant association between children’s sex and food fussiness (t-statistic (1186) = 0.612, *p* > 0.05). There were several significant correlations among the temperament measures. For example, higher impulsivity was correlated with lower inhibitory control fear and shyness while higher fear and shyness were correlated with higher inhibitory control.

### 3.5. Multivariable Regression Analysis Between Food Fussiness and Temperament Variables

A multivariable regression investigated the associations between the temperament variables and food fussiness. The results are summarized in Table 5. Overall, the final model explained 13.1% variance in food fussiness. Block 1 (age) and the temperament variables in Block 2 added significantly to the variance explained. Greater food fussiness was associated with lower impulsivity and lower inhibitory control. Significant associations were not found for shyness and fear (*p* > 0.05). One unit decrease in impulsivity and inhibitory control were associated with 0.41 and 0.75 unit increase in food fussiness, respectively. The interaction variables in Block 3 did not explain additional variance; however, there was a significant interaction between inhibitory control and impulsivity. The final model yielded a moderate effect size (partial eta-squared = 0.137), and the effect size of each variable added to the final model was small (partial eta-squared < 0.02).

**Figure 1:**
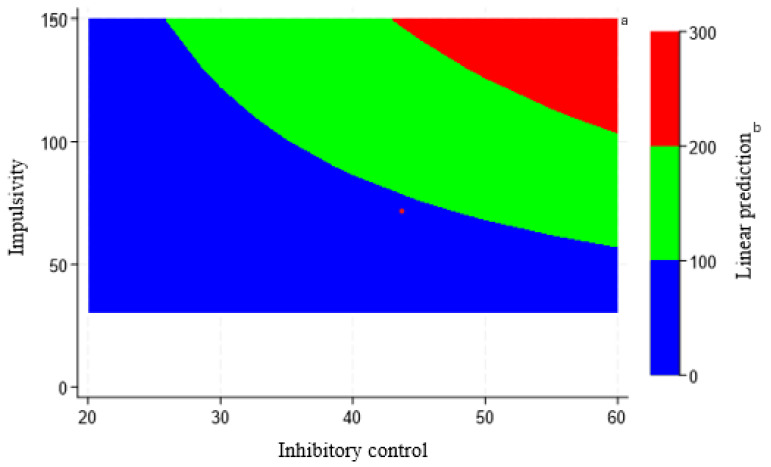
Predicted probability of food fussiness from impulsivity and inhibitory control in 1187 Australian children aged 5 to 12 years. ^a^ Colors in the graph correspond to expected food fussiness for combinations of inhibitory control and impulsivity. The results for impulsivity and inhibitory control were from the 7-point rating scales scale. The contours of the graph demonstrate the interaction of inhibitory control and impulsivity. Inhibitory control and impulsivity have a synergistic interaction to predict food fussiness. ^b^ Indicates linear prediction model predicted values. A linear regression was conducted to assess the predicted probability of food fussiness from impulsivity and inhibitory control with food fussiness as the dependent variables and impulsivity and inhibitory control as independent variables.

To represent the interaction between impulsivity and inhibitory control in the prediction of food fussiness, a two-way contour plot from marginal model prediction was used. Consistent with the regression coefficient, Figure 1 shows that the combination of high impulsivity and high inhibitory control is associated with higher food fussiness. This contrasts with the results for the main effects, where higher food fussiness was associated with lower impulsivity and lower inhibitory control.

## 4. Discussion

The main purpose of the present research was to investigate possible processes associated with food fussiness in children older than early childhood and into middle childhood (here 5-to-12-year-olds) based on a dual-processing approach. The first set of results is from the correlations, the multiple regression main effects and for severely fussy children in the ANOVA analyses. These showed that (a) bottom–up components of higher levels of fear and shyness plus lower impulsivity together with (b) lower inhibitory control as a top–down component were associated with higher fussiness. The second result is from a small but significant interaction in the multiple regression. This suggested that, for some children, food fussiness was associated with higher impulsivity combined with higher inhibitory control. Finally, the results contribute to evidence about the incidence of food fussiness in children after early childhood and into middle childhood.

The first set of results are in accord with the frequent or traditional conceptualization of food fussiness in childhood as arising from food avoidance reactivity that is not regulated by inhibitory control or other top–down processes [43]. The results for inhibitory control imply that children who can respond to involuntary and reflexive avoidance reactions to food and food cues in a more reflective way are likely to be less fussy compared to those less able to regulate their reactions. Lower inhibitory control could be indicative of a general disposition to environmental reactivity that includes novel or disliked foods [43]. These findings are consistent with conclusions that qualities such as effortful control and executive functions are related to the development of food-related self-regulation of both food approach and food avoidance responses [44]. Providing further support for the role of inhibitory control in food fussiness, Reis et al. found that high inhibitory control in 18-month-old Canadian toddlers predicted lower food fussiness in 72-month-old toddlers [45]. Children with better inhibitory control could be more able to accept a variety of healthy foods, while children who are lower in control abilities are less able to accept foods they do not like or do not want to taste [43]. This possibility is supported by evidence that children with higher levels of food neophobia and food pickiness were lower in cognitive flexibility [46].

With respect to possible bottom–up processes, impulsivity refers to a behavioral tendency to respond to external cues in the absence of forethought, planning or self-control [47]. The regression main effects and correlations suggest that impulsive children are less prone to be fussy eaters. Impulsivity has been linked to food approach behaviors and is associated with obesity, and emotional overeating in children [43,44,48]. In this sense, greater impulsivity could be associated with food approach responses. Lower impulsivity, therefore, could be involved in food fussiness via lower food approach responses to novel or different foods.

In relation to other possible bottom–up processes, we found that higher fear and shyness were associated with greater food fussiness. Previous studies have reported similar results, with higher fear being associated with greater food neophobia [32,34,49,50]. Nevertheless, some studies have found no associations between shyness and food fussiness in children [9,33]. Fear and shyness have been found to be associated with behavioral inhibition and therefore could be associated with food avoidance [43,44]. Other studies have reported similar results to the present ones, with higher fear being associated with greater food neophobia [32,34,49,50]. Fear and shyness have also been found to be associated with behavioral inhibition, and this could be a reason it is linked with food avoidance [43,44]. In the present results of regression analysis, however, low impulsivity more than fear and shyness seemed to be a factor in food fussiness.

The interaction between impulsivity and inhibitory control in the prediction of food fussiness accounted for a small amount of variance but nevertheless points to a possible separate dimension of food fussiness in this sample of 5-to-12-year-old children. For some children, it seems that higher impulsivity combined with higher inhibitory control was associated with higher fussiness. Middle childhood, an age including the present sample, is a period when children have increased abilities to control their food environments and so food preferences are likely to become progressively more important. It is also a period when their behavior is increasingly influenced by their own goals and motivation [51], with enhanced abilities in self-regulation and executive functions [52]. The interaction result indicates that at this age, food fussiness for some children could be associated with a bottom–up impulsive rejection of food (“I am not going to eat that”, with little attention to the food itself) together with top–down strategies to control their food environment.

Age has been consistently related to the onset, progress and associations with food fussiness [30,53]. Consistent with the results of Rahill et al. (2019) [30], in their sample of 5-to-12-year-old children, the present finding was that food fussiness was relatively lower for older children (aged 9 to 12 years) compared to younger children (aged 5 to 8 years). In contrast, several studies have not found significant differences in food fussiness as a function of age [54,55]. Clearly, there is scope for further research on age-related differences in food fussiness from infancy through middle childhood and beyond, including individual trajectories [53].

The results showed an overall prevalence of food fussiness to be 43%, composed of 32% severe and 11% moderate fussiness, using the cut-off points developed by Steinsbekk et al. (2017) [38]. This rate is higher than Steinsbekk et al.’s (2017) [38] Norwegian sample of 6-year-old children (*n* = 752), which reported a 26% overall prevalence of fussiness (5% severe and 21% moderate fussiness). The present estimate does however compare well with a similar sample size from another study using the same cut-offs that estimated 47% overall prevalence with 30% severe and 17% moderate fussiness in 1272 4-year-old Swedish children [56]. A variety of prevalence rates have been reported for children up to ages 12 or 13 years [22]. The differences in prevalence rates might be explained by the use of different parent-report instruments as well as behavioral measures, together with associated definitional differences [12]. To allow for more comparability in prevalence rates standardized, reliable and valid fussiness measures appropriate for use in different populations and age groups are needed [22]. In addition, comparisons between parent and self-report measures together with behavioral measures of fussy eating, picky eating and food neophobia (and their associated definitions) would help in clarifying these constructs and their relations [57].

The present findings on food fussiness processes from a dual-processing perspective need to be placed in the context of the wider research on factors associated with fussy/picky eating. Previous research has examined associations between fussy/picky eating and autism spectrum disorders, possibly arising from impaired sensory processing and rigid behavior patterns [58,59]. Children’s eating behaviors and traits such as lower enjoyment of food, higher satiety responsiveness and tactile sensitivity [58] as well as lower cognitive flexibility [46], poorer food categorization abilities [60], and several biologically based characteristics such as leptin level and chemosensory receptor genes [9] could contribute to fussy eating [58]. Children’s decision-making strategies, affective evaluation of food, and disgust sensitivity could also be factors [3]. Parent feeding practices and beliefs have also been related to fussy eating in children [2,58,61]. In a broader theoretical treatment, it could be shown how other influences and correlates impact fussy eating through an effect on either bottom–up reactivity or top–down regulatory control.

There are several limitations of the present study. The parent-report nature of the questionnaires used for both food fussiness and the bottom–up, top–down measures is possibly subject to common method bias [62]. Additionally, while parents know their child the best and are therefore appropriate informants, their ratings could be affected by social desirability bias, such as avoiding an unfavorable characterization of their child, or answering how they think the item should be rated [63]. The possibility of social desirability bias in parent-report and self-report measures, including the CEBQ, the Child Food Rejection Scale and/or reports about eating, diet and health has been recognized as a possible limitation of this approach to measurement [63,64,65,66,67,68,69,70]. For this reason, the use of multiple methods and sources of data, including direct observation and independent assessments in future studies is recommended [65,70]. In addition, research could include a psychometrically validated self-report measure of social desirability in order to assess the possible impact on reports of children’s eating behaviors, including food fussiness [71]. The investigation of processes associated with food fussiness would be strengthened by bottom–up, top–down measures that are more food-related, together with behavioral or laboratory-based measures and self-report (rather than parent-report) measures. Although CBQ is widely used to measure children’s temperament, the questionnaire was designed and validated for 3–7-year-old children and therefore might not be as applicable to the 5–12-year-old participants in the present study. However, the majority of the participants in the present study were in the younger end of the age scale (5–7-year-olds), which is consistent with the age appropriateness of the questionnaire. The bottom–up, top–down measures (fear, shyness, impulsivity, inhibitory control) are general and not specific to food. Lastly, the study participants were children who visited a science museum. This is a selective sample, and limited demographic information was collected. It is therefore unclear how representative it is of food fussiness in the Australian population and the extent to which the results can be generalized. Nevertheless, there do not appear to be reasons to expect that food fussiness processes (the focus of the research) would be biased in this sample.

## 5. Conclusions

The results provide support for value in a dual-processing approach to food fussiness, thereby contributing insights about possible avoidance processes, together with regulatory processes and how they function in children’s food fussiness. Because of the impact of food fussiness on children’s diet quality, it is important to better understand factors and processes associated with food fussiness across childhood. It is important in interpreting children’s food fussiness to take account of both the apparent avoidance response and reflective responses and associated inhibitory control of the avoidance.

A contribution of dual-processing approach is that it specifically draws attention to regulatory processes and abilities in response to avoidance responses to food, including factors and abilities that contribute to these responses, such as food literacy [72], effortful control [15] and the literature on executive function and its development [15,52]. An important additional contribution of the present findings is that they suggest that for some children in middle childhood apparent food fussiness could be related to their use of fussiness to control their food environment. More research is needed with this age group to better understand possible different dimensions of “food fussiness”, such as avoidance and lower regulatory control as one dimension, with child efforts to manage their food environment as another dimension. Understanding these dimensions has implications for approaches to preventive interventions, and for food parenting practices as well as helping parents interpret their children’s food-related behavior.

## Figures and Tables

**Table 1:** Demographic characteristics of 1188 Australian children aged 5 to 12 years participating in the children’s eating and weight study.

Characteristics	Mean ± SD or *n* (%)
Age in years, mean ± SD	7.8 ± 2.0
Age group, *n* (%)	
1. 5–7 years	571 (48.0)
2. 8–9 years	332 (28.0)
3. 10–12 years	285 (24.0)
Sex, *n* (%)	
Males	619 (52.1)
Females	569 (47.9)
BMIz groups, *n* (%)	
1. Underweight	148 (14.3)
2. Healthy weight	671 (65.0)
3. Overweight	155 (15.0)
4. Obese	59 (5.7)

**Table 2:** Prevalence and mean scores of food fussiness groups in 1188 Australian children aged 5 to 12 years.

Fussiness Groups	Prevalence, *n* (%)	Mean Fussiness Score ± SE	*p*-Value
Non-fussy	671 (56.5)	2.2 ± 0.6	<0.001
Moderately fussy	134 (11.3)	3.2 ± 0.1	
Severely fussy	382 (32.2)	4.1 ± 0.5	

**Table 3:** Mean scores of child’s characteristics and temperament among the food fussiness groups and tests for differences.

Variables	Food Fussiness Groups			F-Statistics	*p*-Value
	Non-Fussy (Mean ± SE)	Moderately Fussy (Mean ± SE)	Severely Fussy (Mean ± SE)		
Age, years (*n* = 1188)	8.0 ± 2.0	7.6 ± 2.0	7.7 ± 2.0	-	0.18
BMI (kg/m^2^, *n* = 1033)	17.3 ± 3.2	16.1 ± 2.4	16.6 ± 3.4	F (2, 1031) = 9.68	<0.001
Inhibitory control (*n* = 1187)	5.3 ± 1.0	5.1 ± 1.0	4.9 ± 1.1	F (2, 1184) = 25.13	<0.001
Impulsivity (*n* = 1187)	4.3 ± 1.1	4.2 ± 1.0	4.0 ± 1.2	F (2, 1184) = 5.64	<0.01
Fear (*n* = 1188)	3.8 ± 1.1	4.1 ± 1.1	4.2 ± 1.1	F (2, 1185) = 12.51	<0.001
Shyness (*n* = 1188)	3.5 ± 1.3	3.8 ± 1.3	4.0 ± 1.3	F (2, 1185) = 15.49	<0.001

**Table 4:** Correlations among child characteristics, food fussiness and temperamental qualities in 5–12-year-old Australian children.

Characteristics	Food Fussiness	Age	BMI	Inhibitory Control	Impulsivity	Fear
Age (*n* = 1188)	−0.06 *					
BMI (*n* = 1033)	−0.11 **	0.40 **				
Inhibitory control (*n* = 1187)	−0.26 **	0.08 **	0.04			
Impulsivity (*n* = 1187)	−0.10 **	−0.06 *	0.05	−0.27 **		
Fear (*n* = 1188)	0.19 **	−0.11 **	−0.06 **	−0.05	−0.16 **	
Shyness (*n* = 1188)	0.21 **	−0.02	−0.08 *	0.06 *	−0.52 **	0.28 **

* *p* < 0.05, ** *p* < 0.01.

**Table 5:** Results for multivariable regressions of associations between food fussiness and temperament variables in 1188 Australian children aged 5 to 12 years.

	Coefficient (B)	95% Confidence Interval	Partial Eta-Squared	t-Statistics	*p*-Value	R^2^ Change
Block 1 (Covariate)					<0.001	0.058
Age	−0.01	(−0.04, 0.01)	0.001	−0.93	0.36	
Block 2 (Main effect)					<0.001	0.07
Impulsivity	−0.41	(−0.72, −0.11)	0.007	−2.65	<0.05	
Fear	−0.06	(−0.32, 0.19)	0.000	−0.48	0.63	
Shyness	0.00	(−0.26, 0.26)	0.000	0.03	0.98	
Inhibitory control	−0.75	(−1.16, −0.34)	0.012	−3.53	<0.001	
Block 3 (Interaction effect)					0.1	0.005
Inhibitory control x impulsivity	0.07	(0.01, 0.12)	0.005	2.21	<0.05	
Inhibitory control x shy	0.02	(−0.03, 0.07)	0.001	0.79	0.43	
Inhibitory control x fear	0.03	(−0.02, 0.08)	0.002	1.28	0.2	

## Data Availability

The data presented in this study are available on request from the corresponding author due to ethical reasons.

## Abbreviations

The following abbreviations are used in this manuscript:

BMI	Body mass index
CBQ	Children’s Behavior Questionnaire
CEBQ	Children’s Eating Behavior Questionnaire
